# Submitral Aneurysm in a Patient with a Normal Electrocardiogram

**DOI:** 10.1155/2019/7828909

**Published:** 2019-02-12

**Authors:** Carlos A. Solis-Olivares, Irene Meester, Juan M. Solis-Soto

**Affiliations:** ^1^Instituto Mexicano del Seguro Social, Hospital General de Zona 67, Apodaca, Nuevo León 66600, Mexico; ^2^Universidad de Monterrey, Escuela de Medicina, San Pedro Garza Garcia 66238, Mexico; ^3^Universidad Autonoma de Nuevo Leon, Facultad de Odontologia, Monterrey 64460, Mexico

## Abstract

A ventricular aneurysm entails well-known risks for the patient such as heart failure, potentially lethal arrhythmias, and systemic embolic phenomena. The submitral or posterolateral ventricular aneurysm is a very rare variety, usually of congenital etiology, which may also have other causes, including ischemic heart disease. The present case is about a 76-year-old male with the antecedent of an acute myocardial infarction 3 years ago. He presented with intermittent, brief, and self-limiting episodes of severe dyspnea, intense desperation, and accelerated palpitations, with a nonspecific electrocardiogram. An echocardiography revealed a large submitral aneurysm, with a good clinical response to the specific treatment of heart failure, antiarrhythmics, and oral anticoagulation therapy. We analyze the implications of an aneurysm in the context of an ischemic etiology, with special attention to the limitations of the electrocardiogram in the diagnosis of occlusions of the circumflex artery that irrigates the posterolateral region of the heart. We suspect that a greater number of patients with a culprit circumflex artery could receive appropriate coronary interventionism or thrombolysis if decision-making in the emergency room would not depend mainly on the electrocardiogram. Better stratification tools are needed to prevent late complications of infarction, such as those observed in this patient.

## 1. Introduction

Prior to current modern treatment of acute coronary syndromes, it was estimated that a ventricular aneurysm (VA) developed in 30-35% of patients with acute myocardial infarction (AMI) with an elevated ST-T segment. With the advent of fibrinolysis and coronary angioplasty, the incidence of VA has decreased to 8-15% [1]. The most common etiology of these aneurysms is ischemic heart disease, although there may be other causes.

According to the Mexican Health Secretary, 70-85% of VAs occur in the apical and anterior region of the left ventricle, whereas the lateral wall is the least common location [1]. Also worldwide, VAs most often involve the apex (31.7%), the posterior wall (23.8%), and the anterior wall (9.5%), but only 1% of cardiac aneurysms affect the lateral region of the left ventricle and these aneurysms tend to be small-sized [2].

The clinical importance of VA ranges from asymptomatic to potentially lethal ventricular arrhythmias; sometimes, there are signs and symptoms of heart failure or episodes of arterial embolism.

We present the case of a septuagenarian male patient who was evaluated at a private cardiology clinic because of frequent and severe paroxysmal symptoms of heart failure and palpitations, which were strikingly brief and self-limiting, with nonspecific findings in the electrocardiogram (ECG). Finally, a VA with an unusual submitral or posterolateral location was detected by echocardiography.

## 2. Case

A 76-year-old Mexican man, from the state of San Luis Potosí, Mexico, was examined at a private cardiology clinic in the Mexican state of Nuevo Leon in April 2018 because of episodes of excessive dry cough, severe dyspnea, and accelerated palpitations as of the previous day. These episodes were frequent and of sudden onset and short duration, without angina, lipothymia, or syncope. He was asymptomatic between episodes.

There were no nonpathological antecedents of importance; he had worked as a farmer until his retirement; he did not smoke nor consume alcohol or drugs; and he was neither diabetic nor hypertensive. The only personal pathological antecedent of interest was a hospitalization due to an AMI 3 years ago, which had been attended in the patient's native state. Access to his medical file was not an option at the moment of the consult.

Upon arrival at the clinic, he was calm and symptom-free. However, at the start of the clinical interview, he suddenly presented a new episode of severe dyspnea accompanied by intense desperation, which lasted less than 1 minute, after which he remained calm and asymptomatic. The physical examination did not yield relevant data, except for arrhythmic heart sounds due to premature beats. The pulmonary fields were considered clean and well-ventilated, abdominal visceromegalies were not found, and no edema was detected in the lower limbs. The blood pressure was 120/80 mmHg, the heart rate 76 bpm, and the respiratory rate 20 respirations per minute.

**Figure 1:**
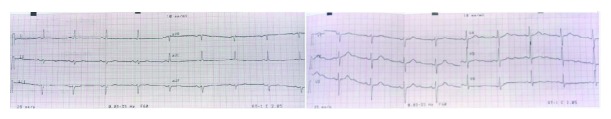
Admission ECG without abnormalities.

**Figure 2:**
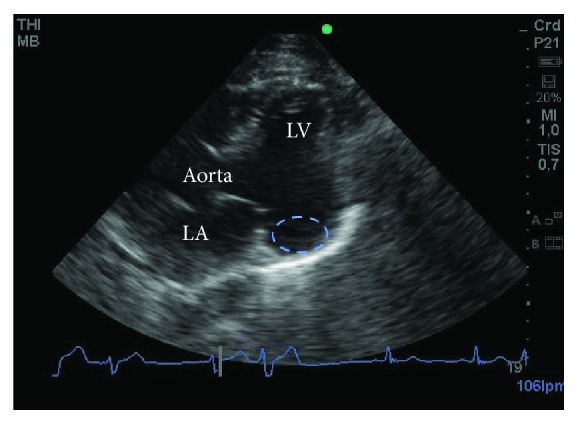
Transthoracic echocardiogram, parasternal long axis view. Notice the large submitral aneurysm (diameter, 3 cm) in the posterolateral basal-medial region of the left ventricle (encircled area). LA: left atrium; LV: left ventricle.

**Figure 3:**
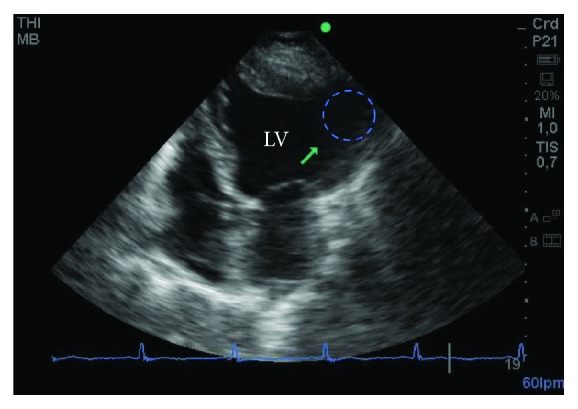
The echocardiogram reveals a large aneurysm (diameter, 3 cm; encircled area) in the lateral basal-medial region of the ventricle without apparent thrombus (arrow). LV: left ventricle.

The initial ECG revealed a sinus rhythm with a heart rate of 55 bpm, PR 0.18, QRS 0.08, AQRS at -30 degrees, and tracing without significant abnormalities (Figure 1). A ventricular arrhythmia was suspected, but a 24 h Holter monitoring was discarded because of the apparent urgency of the situation. Instead, a color Doppler echocardiography was performed immediately. The echocardiogram revealed a left ventricular ejection fraction (LVEF) of 34% and a large submitral aneurysm in the posterolateral basal-medial region of the left ventricle, as well as severe anterior and inferior basal-medial hypokinesia, mild mitral valve insufficiency, and no pulmonary artery hypertension (Figures 2 and 3).

As the patient presented symptoms of intermittent heart failure probably due to paroxysmal tachycardia, which, in the context of a cardiac posterolateral submitral VA (SVA), could be potentially lethal, medical treatment was initiated with spironolactone (50 mg/day), furosemide (20 mg/day), amiodarone (200 mg every 12 hours for 2 weeks and then a single 200-mg dose daily), nitroglycerin patches (5 mg/day), and apixaban (5 mg every 12 hours, orally).

**Figure 4:**
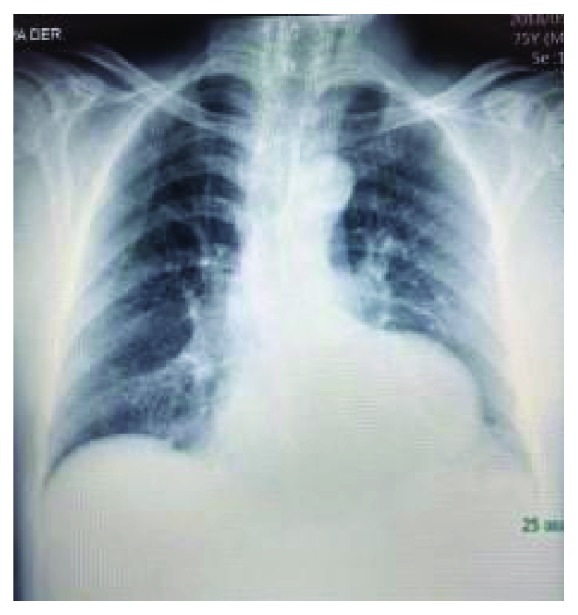
Posteroanterior thoracic radiography denotes cardiomegaly imparted mostly by the low left cardiac bulging.

On a control visit three weeks later, the patient had no cardiovascular symptoms (New York Heart Association (NYHA) functional class I) and had not experienced recurrence of palpitations nor episodes of sudden dyspnea. The laboratory blood values were as follows: hemoglobin, 16.4 gr/dL; leukocytes, 7,400/mm^3^; platelets, 261,000/mm^3^; glucose, 108 mg/dL; creatinine, 1.16 mg/dL; uric acid, 5.5 mg/dL; total cholesterol, 207 mg/dL; triglycerides, 147 mg/dL; low-density lipoprotein cholesterol (LDL-c), 144.3 mg/dL; and high-density lipoprotein cholesterol (HDL-c), 51 mg/dL. Hepatic function tests and serum electrolytes were normal, except for an elevated thyroid-stimulating hormone (TSH) level (45 mIU/mL). Urinalysis was without significant abnormalities: pH, 7.5 and density, 1.010. The posteroanterior chest radiograph (Figure 4) revealed grade II cardiomegaly with a rounded growth on a left profile, compatible with VA, without further abnormalities.

Levothyroxine (first week, 50 *μ*g/day; from second week onwards, 75 *μ*g/day) and simvastatin (20 mg/day) were added to the treatment. With this therapeutic scheme, the patient remained asymptomatic until August 2018, month of the last follow-up visit at this private clinic. At the beginning of this month, the last monitored TSH level was 7.48 mUI/mL. The good clinical response to treatment and the tendency to a sinus bradycardia did not seem to justify the inclusion of beta-blockers.

The patient, with low economic resources, will continue his follow-up and treatment at a third-level public hospital in Monterrey, Mexico. Cardiac gammagraphy, cardiac catheterization, and electrophysiological monitoring were recommended.

## 3. Discussion

SVA is a peculiar pathology, which is rarely detected worldwide. The majority of cases occur in the African population and in a lesser proportion in the Indian population [3, 4]. SVA can be defined as a dilation of the ventricle and a thinning of the ventricular wall below the mitral valve apparatus, due to a weak union of the cardiac muscle to the fibrous tissue adjacent to the posterolateral mitral valve. Most often, there is a congenital etiology, but any of the following may be an alternative cause: infection, for example, tuberculosis and endocarditis; inflammation as in rheumatic fever or Takayasu disease; neoplasia; post-AMI; and idiopathic causes [5, 6]. Patients with this problem may be asymptomatic or have symptoms of heart failure, palpitations, lipothymia, syncope, or sudden death, in addition to systemic embolic phenomena. The ECG often shows nonspecific findings, unlike VA cases due to ischemia of the apical or anterior wall, which are detected by a persistent ST-T segment elevation and the presence of Q waves in electrocardiographic derivations corresponding to the affected area. In fact, localization of aneurysms in the posterolateral region of the left ventricle, as in the present case, is very rare.

The initial objective when detecting a SVA is to corroborate an ischemic etiology and rule out other potential causes.The therapy goal is to offer interventional or surgical treatment of coronary vessels with critical lesions and/or myocardial ischemia and viability, resection of the SVA, or, if surgery seems too risky, a cardioverter defibrillator implant.

Considering that this case concerns a senile patient with a clinical history of hospitalization due to an AMI 3 years ago, combined with the echocardiographic findings of segmental alterations of left ventricle mobility in regions other than the VA area, it seems most probable that ischemia had caused this SVA, although a congenital or other origin cannot be ruled out completely. It is known that 40% to 50% of the occlusions of the circumflex left coronary artery (LCA) in the context of myocardial infarction do not alter the ST-T segment in the ECG [7]. The ECG, therefore, is insufficient to detect a narrowing or occlusion of the LCA and can lead to a wrong classification of risk and a delay in the performance of coronary angiography and interventional procedures [8]. This situation probably applies to this case. Patients with an AMI due to an occluded LCA tend to have a worse evolution than patients with an occluded right coronary artery. Among other reasons, this could be because of a delay in the establishment of fibrinolytic or interventional reperfusion therapy in cases with an ECG with nonspecific findings, which is more common when the culprit occlusion occurs in the LCA [9]. Finally, supposing that the posterolateral SVA of this case is due to ischemia, the issue that deserves attention is the inadequate procedure of risk stratification of emergency room patients with chest pain and a suspect of cardiac ischemia. Up to 10% of patients with thoracic pain and the suspicion of cardiac ischemia are dismissed because of a normal ECG but return within 24 to 48 hours with an established AMI and are no longer within the therapeutic window for fibrinolytic or interventional reperfusion therapy [10].

In clinical practice, the LCA is the least frequent culprit vessel among patients treated invasively for AMI. Patients with LCA occlusion may present an acute non-ST segment elevation myocardial infarction and require emergency coronary intervention [11]. The consequences of this inadequate risk stratification, based mainly on a normal ECG but without considering an adequate semiology of chest pain, which should include studies such as troponin levels, echocardiography, and nuclear medicine studies, can lead to a substantial delay in reperfusion therapy and the appearance of late complications such as VA.

## 4. Conclusion

SVA, usually congenital in origin and peculiar for its rare occurrence and particular anatomical location in the heart, may also be of ischemic origin. Occlusions of the circumflex artery as a culprit lesion of myocardial infarction, often invisible in an ECG, could lead to the development of VA in elderly patients whom were not treated timely with coronary reperfusion therapy. This case demonstrates the need to improve clinical risk categorization strategies in patients evaluated for chest pain in an emergency service.
